# Accuracy of ultrasonographic changes during neoadjuvant chemotherapy to predict axillary lymph node response in clinical node-positive breast cancer patients

**DOI:** 10.3389/fonc.2022.845823

**Published:** 2022-07-22

**Authors:** Zhuoxuan Li, Yiwei Tong, Xiaosong Chen, Kunwei Shen

**Affiliations:** Department of General Surgery, Comprehensive Breast Health Center, Ruijin Hospital, School of Medicine, Shanghai Jiao Tong University, Shanghai, China

**Keywords:** breast cancer, lymph nodes, ultrasound, nomogram, neoadjuvant chemotherapy

## Abstract

**Purpose:**

To evaluate whether changes in ultrasound features during neoadjuvant chemotherapy (NAC) could predict axillary node response in clinically node-positive breast cancer patients.

**Methods:**

Patients with biopsy-proven node-positive disease receiving NAC between February 2009 and March 2021 were included. Ultrasound (US) images were obtained using a 5-12-MHz linear array transducer before NAC, after two cycles, and at the completion of NAC. Long and short diameter, cortical thickness, vascularity, and hilum status of the metastatic node were retrospectively reviewed according to breast imaging-reporting and data system (BI-RADS). The included population was randomly divided into a training set and a validation set at a 2:1 ratio using a simple random sampling method. Factors associated with node response were identified through univariate and multivariate analyses. A nomogram combining clinical and changes in ultrasonographic (US) features was developed and validated. The receiver operating characteristic (ROC) and calibration plots were applied to evaluate nomogram performance and discrimination.

**Results:**

A total of 296 breast cancer patients were included, 108 (36.5%) of whom achieved axillary pathologic complete response (pCR) and 188 (63.5%) had residual nodal disease. Multivariate regression indicated that independent predictors of node pCR contain ultrasound features in addition to clinical features, clinical features including neoadjuvant HER2-targeted therapy and clinical response, ultrasound features after NAC including cortical thickness, hilum status, and reduction in short diameter ≥50%. The nomogram combining clinical features and US features showed better diagnostic performance compared to clinical-only model in the training cohort (AUC: 0.799 vs. 0.699, P=0.001) and the validation cohort (AUC: 0.764 vs. 0.638, P=0.027).

**Conclusions:**

Ultrasound changes during NAC could improve the accuracy to predict node response after NAC in clinically node-positive breast cancer patients.

## Introduction

According to the latest global cancer statistics, breast cancer has become the most commonly diagnosed cancer in women with 2.3 million new cases in 2020 (1). Axillary lymph node status is one of the most important factors for clinical staging and also an independent prognostic predictor for breast cancer patients (2). Neoadjuvant chemotherapy (NAC) has become an important treatment for inoperable locally advanced and large operable breast cancer patients (3). NAC aims to convert inoperable breast tumors to operable disease and to downstage the primary large breast tumor and metastatic axillary lymph node. According to previous reports, 40%-60% of node-positive patients could convert to node-negative after NAC. Predictive markers of candidates who could benefit from NAC such as molecular subtype, NAC regimens, and clinical response had been extensively confirmed (3–5). However, accurate evaluation of the response to NAC remains to be investigated.

For patients with primary node-positive disease, axillary lymph node dissection (ALND) remains the standard of care after completion of NAC (6). However, ALND is usually followed by increased risk of complications, including lymphedema and paresthesia, which leads to poor quality of life (7). To avoid such complications, the possibility of sentinel lymph node biopsy (SLNB) for patients achieving pCR after completion of NAC with primary node-positive disease was evaluated by the ACOSOG (American College of Surgeons Oncology Group) Z1071 and SENTINA (sentinel lymph-node biopsy in patients with breast cancer before and after neoadjuvant chemotherapy) trials and the false-negative rates (FNR) of SLNB in these two randomized trials were 12.6% and 14.2%, which were found to be above the acceptable 10% cut point. Although a reduced FNR of less than 10% could be achieved by using dual tracer or removing at least three sentinel lymph nodes (8, 9), SLNB remains debatable with relatively FNR for patients presenting clinically node-negative after completion of NAC according to the guideline (10). Predictive markers to select axillary pCR patients appropriate for SLNB is still a challenge.

Imaging modalities have been applied to increase the diagnostic accuracy for lymph node response evaluation. For instance, when axillary ultrasound (US) was added to assess the axillary response after NAC, the FNR decreased to 9.8% in the ACOSOG Z1071 population (11). A previous study has demonstrated that axillary lymphadenopathy in US after NAC had the strongest predictive capacity of residual axillary LN metastasis (OR=13.8), while other clinical predictive features including clinical N stage, Ki-67 negativity, hormone receptor positivity, and HER2 negativity showed an OR from 2.3 to 3.7 (12, 13). Moreover, breast pCR was also an independent positive predictor for nodal response in the Z1071 trial (14). Likewise, as demonstrated in our previous study, patients with breast pCR had a significantly lower ypN+ rate than those with residual tumor (23.9% vs. 62.5%, OR=0.14) (15). US features of lymph nodes observed after chemotherapy, including shorter short-axis, shorter long-axis, hilum preservation, and absence of cortical thickness, have been proven to be associated with axillary pCR (16, 17). In addition to the observation of the lymph node status at a certain point in time, US also has the advantages of convenient, dynamic, and continuous observation throughout the treatment (16, 18). In one study, axillary response was evaluated at separate time points before, during, and after NAC, and the results showed that only mid-NAC US features including breast tumor size and cortical thickness showed an average diagnostic performance with an AUC of 0.760 (19). Although different time points were included in this study, the imaging change of lymph nodes across treatment cycles, which might reflect treatment response, was not investigated. To further understand the association between the specific lymph node US features throughout the treatment and ALN response after NAC, more markers combining different time points of lymph node specific US indicators is needed to be explored.

Therefore, the aim of our study was to evaluate whether changes of ultrasound features during neoadjuvant chemotherapy (NAC) could predict axillary node response in clinically node-positive breast cancer patients, thus to develop a novel nomogram combining clinical and axillary US features to predict the probability of axillary nodal pCR after NAC in primary node-positive patients, which may guide further ALN management after NAC.

## Materials and methods

### Data source and patients selection

We retrospectively reviewed consecutive female patients diagnosed with primary invasive breast cancer who received NAC from February 2009 to March 2021 in Comprehensive Breast Health Center, Shanghai Jiaotong University School of Medicine affiliated Ruijin Hospital. Eligible patients were women with node-positive disease confirmed by fine-needle aspiration biopsy/core needle biopsy before NAC initiation and with US monitoring of axilla performed at baseline, after two cycles, and after completion of NAC (**Supplementary Figure S1**). The exclusion criteria were as follows (1): without biopsy-proven nodal metastases (n=163) (2); absence of US images before, during, or after NAC (n=146) (3); treated with neoadjuvant endocrine therapy alone (n=9). The current study was approved by the independent Ethical Committees of Ruijin Hospital, Shanghai Jiaotong University School of Medicine.

### Clinical and pathological evaluation

Patient clinical and pathological data were retrieved from Shanghai Jiaotong University Breast Cancer Database (SJTU-BCDB). Core needle biopsy and fine needle aspiration biopsy were performed for suspicious breast and lymph node lesions. Pathological evaluation was performed at the Department of Pathology, Ruijin Hospital by at least two independent pathologists. Histological type and pathological grade were referred to the World Health Organization classification (20). Clinical TNM staging was defined according to the Eighth edition of the American Joint Committee on Cancer staging system (21). ER, PR, HER2, and Ki-67 expression were assessed by immunohistochemistry (IHC) methods in core needle biopsy samples at baseline. Samples with HER2 IHC 2+ were further examined by fluorescence *in situ* hybridization (FISH). The positivity criteria accorded to the 2018 American Society of Clinical Oncology/College of American Pathologists (ASCO/CAP) guidelines (22). Molecular subtypes were classified as four types: Luminal A (ER+, PR high, HER2-, Ki67 low), Luminal B (ER+ or/and PR -/low or Ki67 high), HER2-enriched (ER-, PR-, HER2+), and TNBC (ER-, PR-, HER2-) (23). Patients were recommended with NAC after a multidisciplinary discussion (MDT) with surgical oncologist, medical oncologist, radiation oncologist, and other related clinicians. NAC regimes were classified based on anthracycline (A) and taxane (T). Patients were classified into A+T, such as EC-T (epirubicin 90 mg/m^2^ and cyclophosphamide 600 mg/m^2^ followed by docetaxel 100 mg/m^2^ q3w), TEC (docetaxel 75 mg/m^2^, epirubicin 75 mg/m^2^ and cyclophosphamide 500 mg/m^2^ q3w), A (anthracycline)-containing, such as EC (epirubicin 90 mg/m^2^ and cyclophosphamide 600 mg/m^2^ q3w), or T (taxane)-containing, such as PCb (weekly paclitaxel 80 mg/m^2^ and carboplatin AUC 2). Neoadjuvant HER2-targeted therapy based on trastuzumab (8 mg/Kg at first cycle and followed by 6 mg/Kg q3w or 4 mg/Kg at first cycle and followed by 2 mg/Kg weekly) was also applied to patients according to the MDT decision.

After completion of NAC, clinical and pathological evaluations were repeated in the radical surgery specimen. Clinical response was judged according to RECIST 1.1 criteria as CR (complete response, disappearance of all target lesions), PR (partial response, the sum of diameters of target lesions decreased at least 30%), PD (progressive disease, the sum of diameters of target lesions increased at least 20%), and SD (stable disease, neither PD nor PR) (24). The primary endpoint of the current study was nodal complete response, which was defined as no metastatic carcinoma in the axillary lymph nodes (25). Isolated tumor cells or micrometastasis in the nodes were not considered complete response (26).

### US evaluation procedure

Breast and axilla US examinations were performed before NAC (at baseline, before biopsy), after two cycles of NAC, and completion of NAC by experienced radiologists with more than 10 years of experience in breast imaging per individual in the Department of Ultrasonography, Ruijin Hospital. All patients were assessed with real-time US using a 5-12-MHz linear array transducer (Esaote MyLab 60, Esaote SpA, Genoa, Italy). The largest biopsy-confirmed positive node was viewed as the target lesion. US features for analysis included long diameter, short diameter, cortical thickness, vascularity (rare, minimal, or abundant), and hilum (preserved, partially preserved, or completely obliterated) according to the breast imaging-reporting and data system (BI-RADS) (27), as presented in **Supplementary Figure S2**. Imaging reports were retrospectively reviewed from SJTU-BCDB and analyzed in the current study. Changes of the US features were evaluated as the reduction in diameter compared to the baseline. A reduction of 30% in diameter at two cycles, as well as a reduction of 50% (1-0.7*0.7 = 0.51) at completion were applied as cut-offs according to the RECIST 1.1 criteria, where a reduction of 30% in diameter was considered PR (24).

### Statistical analyses

The included population was randomly divided into a training set and a validation set at a 2:1 ratio using simple random sampling method. Categorical variables were analyzed by using Chi-square test or Fisher’s exact test, if necessary. Continuous variables were analyzed by using independent *t*-test and Mann–Whitney *U*-test. Univariate and multivariate binary logistic regression analyses were used to identify the factors associated with axillary pCR in the training set.

Receiver operator characteristic (ROC) curve was used to assess the diagnostic performance of clinical and US imaging features. The area under the curve (AUC) was obtained at the cut-off value yielding the largest Youden index and compared using generalized estimating equations and the Delong test. Calibration was assessed by calibration plot with 1000 bootstrap resampling. *P <*0.05 was considered to indicate a statistically significant difference. Statistical analysis was performed using SPSS (version 24.0) and R software (version 4.0.5).

## Results

### Baseline patient characteristics

Baseline characteristics of the 296 participants in the training and validation set are described in **Table 1**. Among the 296 patients included, 108 (36.5%) achieved axillary pCR while 188 (63.5%) had residual axillary lymph nodes. No significant difference was observed at baseline between the training set and validation set, which justified their use as two independent sets. The average age of patients was 50 ± 11.9 years. In the training set, the ALN pCR group showed a higher proportion of ER negative (60.0%, *P*<0.001), PR negative (75.0%, *P*=0.002), and HER2 positive (52.3%, *P*=0.001) disease. Patients receiving NAC T-containing regimen (57.6%, *P*=0.006) and neoadjuvant HER2-targeted therapy (50.8%, *P*<0.001) were more likely to achieve ALN pCR. The rate of ALN pCR ranged from 71.4% to 9.1% among patients who had clinical CR and PD.

**Table 1:** Baseline characteristics for the training set and the validation set.

characteristics	Training set			Validation set		
	ypN0n=65	ypN+n=131	P	ypN0n=4	ypN+n=57	P
Age (mean ± SD)	49.63 ± 11.14	50.08 ± 11.73	0.796	51.70 ± 13.17	51.56 ± 12.28	0.958
Palpable node			0.917			0.891
No	12 (18.5%)	25 (19.1%)		8 (18.6%)	10 (17.5%)	
Yes	51 (81.5%)	106 (80.9%)		35 (81.4%)	47 (82.5%)	
cT			0.483			0.873
1	12 (18.8%)	26 (19.8%)		9 (21.4%)	10 (17.9%)	
2	44 (68.8%)	76 (58.0%)		25 (59.5%)	37 (66.1%)	
3	4 (6.3%)	19 (14.5%)		3 (7.1%)	5 (8.9%)	
4	3 (4.7%)	8 (6.1%)		3 (7.1%)	3 (5.4%)	
x	1 (1.6%)	2 (1.5%)		2 (4.8%)	1 (1.8%)	
cN			0.503			0.896
1	37 (56.9%)	65 (49.6%)		25 (58.2%)	31 (54.3%)	
2	24 (36.9%)	51 (38.9%)		17 (39.5%)	23 (40.4%)	
3	4 (6.2%)	15 (11.5%)		1 (2.3%)	3 (5.3%)	
Histology			0.793			0.076
IDC	63 (96.9%)	126 (96.2%)		40 (93.0%)	57 (100.0%)	
Others	2 (3.1%)	5 (3.8%)		3 (7.0%)	0 (0.0%)	
Grade			0.734			0.838
I-II	19 (29.2%)	50 (38.2%)		12 (27.9%)	22 (38.6%)	
III	27 (41.5%)	63 (48.1%)		18 (41.9%)	30 (52.6%)	
NA	19 (29.2%)	18 (13.7%)		13 (30.2%)	5 (8.8%)	
ER			<0.001			0.034
Negative	39 (60.0%)	42 (42.3%)		25 (58.1%)	21 (36.8%)	
Positive	26 (40.0%)	89 (57.7%)		18 (41.9%)	36 (63.2%)	
PR			0.002			0.049
Negative	49 (75.0%)	68 (51.9%)		33 (76.7%)	33 (57.9%)	
Positive	16 (25.0%)	63 (48.1%)		10 (23.3%)	24 (42.1%)	
HER2			0.001			0.013
Negative	31 (47.7%)	94 (71.8%)		18 (41.9%)	38 (66.7%)	
Positive	34 (52.3%)	37 (28.2%)		25 (58.1%)	19 (33.3%)	
Molecular subtype			0.004			0.021
Luminal A	2 (3.1%)	8 (6.1%)		0 (0.0%)	4 (7.0%)	
Luminal B	25 (38.5%)	81 (61.8%)		18 (41.9%)	32 (56.1%)	
HER2 enriched	22 (33.8%)	21 (16.0%)		12 (27.9%)	11 (19.3%)	
TNBC	16 (24.6%)	21 (16.0%)		13 (30.2%)	10 (17.5%)	
Ki-67			0.270			0.109
< 14%	5 (7.7%)	17 (13.0%)		2 (4.7%)	8 (14.3%)	
≥ 14%	60 (92.3%)	114 (87.0%)		41 (95.3%)	48 (85.7%)	
NAC regimen			0.006			0.028
A containing	4 (33.3%)	8 (66.7%)		1 (33.3%)	2 (66.7%)	
T containing	19 (57.6%)	14 (42.4%)		17 (65.4%)	9 (34.6%)	
A+T	42 (27.8%)	109 (72.2%)		25 (35.2%)	46 (64.8%)	
Neoadjuvant HER2-targeted therapy			<0.001			0.048
No	34 (25.2%)	101 (74.8%)		21 (35.0%)	39 (65.0%)	
Yes	31 (50.8%)	30 (49.2%)		22 (55.0%)	18 (45.0%)	
Clinical response			0.003			0.195
CR	10 (15.4%)	4 (3.1%)		5 (11.6%)	2 (3.5%)	
PR	47 (72.3%)	93 (71.0%)		32 (74.4%)	39 (68.4%)	
SD	7 (10.8%)	23 (17.6%)		5 (11.6%)	14 (24.6%)	
PD	1 (1.5%)	11 (8.4%)		1 (2.3%)	2 (3.5%)	

ypN0, nodal pathological complete response; ypN+, residual nodal disease; SD, standard deviation; IDC, infiltrating ductal carcinoma; NA, not available; ER, estrogen receptor; PR, progesterone receptor; HER2, human epidermal growth factor 2; TNBC: triple negative breast cancer; A, anthracycline; T, taxanes; CR, complete response; PR, partial response; SD, stable disease; PD, progressive disease.

### Ultrasound features

US features of the biopsy-confirmed metastatic axillary lymph node are shown in **Table 2**. At baseline, no significant difference in US features was observed between pCR and non-pCR groups (all *P*>0.050). After two cycles of NAC, medians of long diameter (15.6mm *vs*. 18.9mm, *P*=0.041), short diameter (7.3mm *vs*. 9.0mm, *P*=0.013), and cortical thickness (4.2mm *vs*. 5.4mm, *P*=0.011) were shorter in the pCR group compared with the non-pCR group, while vascularity (*P*=0.739) and hilum (*P*=0.270) remained similar. After completion of NAC, medians of long diameter (11.0mm *vs*. 15.6mm, *P*=0.006), short diameter (5.3mm *vs*. 7.1mm, *P*=0.001), and cortical thickness (3.0mm *vs*. 3.6mm, *P*=0.005) were significantly decreased in the pCR group, and hilum preservation was more common (65.4%) in the pCR group compared to the non-pCR group. Abundant vascularity tended to be more observed in the ypN+ population (11.1% vs. 1.9%, *P*=0.052).

**Table 2:** Ultrasound features of biopsy-confirmed metastatic axillary lymph node(s)^a^ of the training set.

	Baseline			2 cycles			Completion		
	yPN0n=65	yPN+n=131	P	ypN0n=65	ypN+n=13	P	ypN0n=65	yPN+n=131	p
Long diameter (mm)	25.0 (17.7-32.8)	25.0 (18.1-32.0)	0.951	15.6 (12.0-22.9)	18.9 (13.2-28.0)	0.041	11.0 (6.8-16.7)	15.6 (9.6-21.3)	0.006
Short diameter (mm)	13.0 (10.0-17.1)	13.0 (9.5-17.0)	0.831	7.3 (5.5-10.0)	9.0 (6.5-11.4)	0.013	5.3 (3.4-7.2)	7.1 (4.8-9.3)	0.001
Cortical thickness (mm)	7.9 (5.9-10.8)	8.5 (5.5-11.2)	0.835	4.2 (3.1-6.2)	5.4 (3.6-7.1)	0.011	3.0 (0.7-4.1)	3.6 (2.6-5.5)	0.005
Vascularity*			0.535			0.739			0.052
Rare	20 (30.8%)	44 (33.6%)		31 (48.4%)	65 (49.6%)		33 (63.5%)	60 (51.3%)	
Minimal	39 (60.0%)	69 (52.7%)		26 (40.6%)	48 (36.6%)		18 (34.6%)	44 (37.6%)	
Abundant	6 (9.2%)	18 (13.7%)		7 (11.0%)	18 (13.7%)		1 (1.9%)	13 (11.1%)	
Hilum*			0.486			0.270			0.005
Preserved	30 (46.2%)	56 (42.7%)		35 (54.7%)	56 (42.7%)		34 (65.4%)	54 (46.2%)	
Partially preserved	17 (26.2%)	45 (34.4%)		13 (20.3%)	37 (28.2%)		16 (30.8%)	35 (29.9%)	
Completely obliterated	18 (27.7%)	30 (22.9%)		16 (25.0%)	38 (29.0%)		2 (3.8%)	28 (23.9%)	

a The largest reported node on ultrasound was chosen as the target lesion.

^*^One patient achieved nodal pCR during NAC; twenty-seven patients achieved nodal pCR after NAC.

The changes of US features for biopsy-confirmed metastatic axillary lymph node were evaluated in the training set (**Table 3**). Patients with ALN pCR tended to show more reduction in lymph node US quantitative features, reduction in cortical thickness ≥30% after two cycles of NAC (69.2%, *P*=0.034), reduction in short diameter ≥50% (69.2%, *P*<0.001), and cortical thickness ≥50% (76.9%, *P*<0.001) after completion of NAC were associated with axillary pCR.

**Table 3:** Changes of ultrasound features for biopsy-confirmed metastatic axillary lymph node during NAC^a^ in the training set.

Characteristics	All n=196	yPN0 n=65	yPN+ n=131	P
After 2 cycles of NAC				
Reduction in long diameter ^b^				0.076
< 30%	134 (68.4%)	39 (60.0%)	95 (72.5%)	
≥ 30%	62 (31.6%)	26 (40.0%)	36 (27.5%)	
Reduction in short diameter				0.077
< 30%	102 (52.0%)	28 (43.1%)	74 (56.5%)	
≥ 30%	94 (48.0%)	47 (56.9%)	57 (43.5%)	
Reduction in cortical thickness				0.034
< 30%	81 (41.3%)	20 (30.8%)	61 (46.6%)	
≥ 30%	115 (58.7%)	45 (69.2%)	70 (53.4%)	
After completion of NAC				
Reduction in long diameter ^c^				0.091
< 50%	116 (59.2%)	33 (50.8%)	83 (63.4%)	
≥ 50%	80 (40.8%)	32 (49.2%)	48 (36.6%)	
Reduction in short diameter				<0.001
< 50%	96 (49.0%)	20 (30.8%)	76 (58.0%)	
≥ 50%	100 (51.0%)	45 (69.2%)	55 (42.0%)	
Reduction in cortical thickness				<0.001
< 50%	82 (41.8%)	15 (23.1%)	67 (51.1%)	
≥ 50%	114 (58.2%)	50 (76.9%)	64 (48.9%)	

a The largest reported node on ultrasound was chosen as the target lesion.

b Change compared to baseline. The cut-off of 30% was set according to the RECIST 1.1 criteria, where a reduction of 30% in diameter was considered partial response.

c Change compared to baseline. The cut-off of 50% was set according to the RECIST 1.1 criteria, where a reduction of 30% in diameter was considered partial response, 50% referred to a reduction of 30% in diameter after two cycles of NAC, and another reduction of 30% in diameter compared to two-cycle after completion of NAC (1-0.7*0.7 = 0.51).

### Univariate and multivariate analysis of predictors for axillary pCR

In the univariate analysis, clinical features including ER status (*P*<0.001), PR status (*P*=0.002), HER2 status (*P*=0.001), molecular subtype (*P*=0.005), clinical response (*P*=0.010), NAC regimen (*P*=0.006), as well as neoadjuvant HER2-targeted therapy (*P*<0.001) were associated with axillary pCR rate in the training set (**Figure S3**). Among US features after two cycles of NAC, short diameter (*P*=0.022), cortical thickness (*P*=0.015), and reduction in cortical thickness (*P*=0.036) were associated with axillary pCR. Among US features after completion of NAC, short diameter (*P*=0.003), cortical thickness (*P*=0.004), hilum status (*P*=0.016), reduction in short diameter ≥50% (*P*<0.001), and reduction in cortical thickness (*P*<0.001) were associated with axillary pCR.

**Figure 1:**
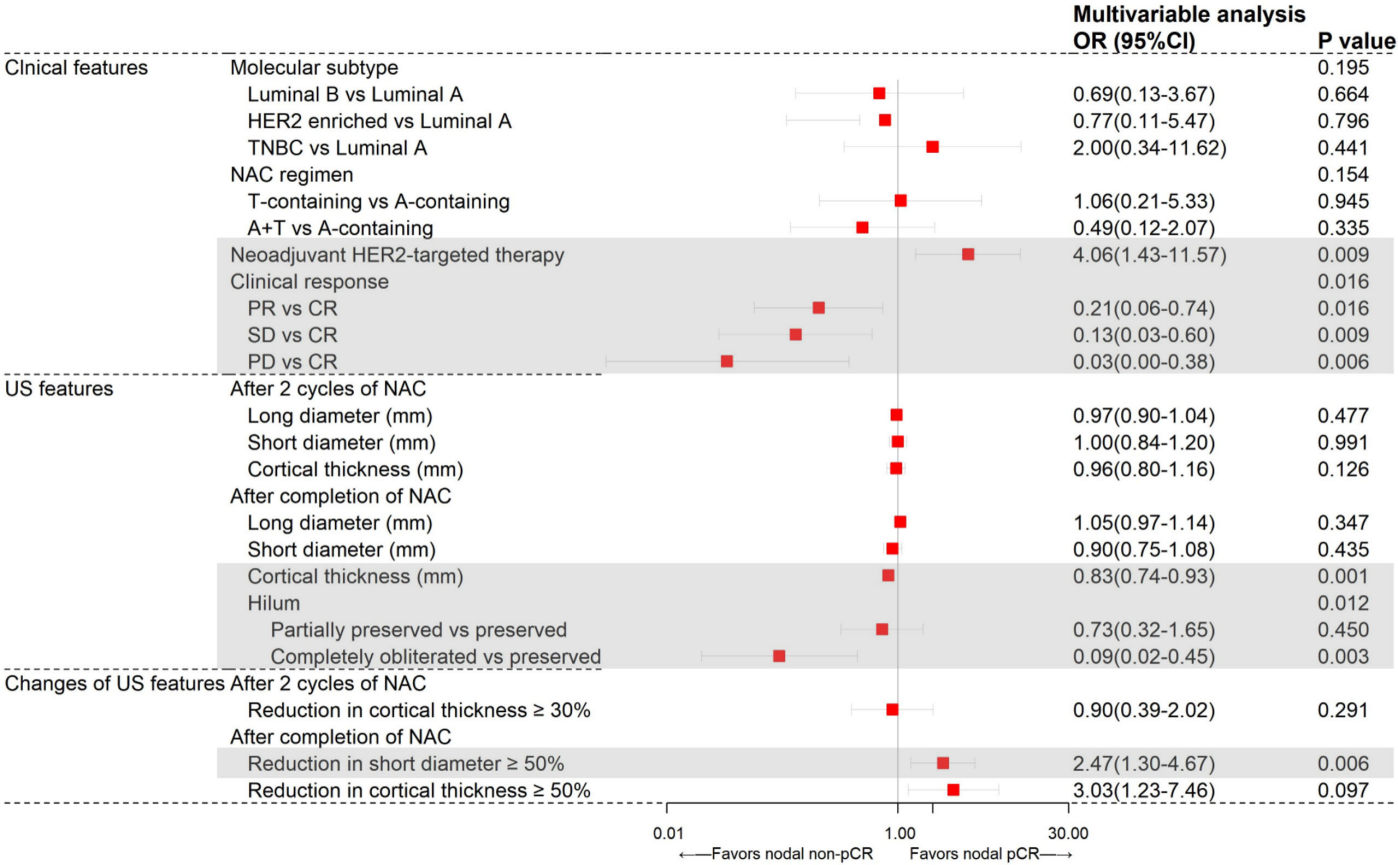
Results from multivariate logistic regression analysis of different variables predicting axillary pCR in the training set (N=196).

In further multivariate logistic regression analysis, neoadjuvant HER2-targeted therapy (*P*=0.009), clinical response (*P*=0.016), US features after completion of NAC including cortical thickness (*P*=0.001), hilum status (*P*=0.012), and reduction in short diameter ≥50% (*P*=0.006) were independent predictors for axillary pCR (**Figure 1**). Patients receiving neoadjuvant HER2-targeted therapy (OR=4.06, 95%CI 1.43-11.57, *P*=0.009) were more likely to achieve nodal pCR. Patients who had PR (OR=0.22, 95%CI 0.06-0.75, *P*=0.016), SD (OR=0.13, 95%CI 0.03-0.60, *P*=0.009), and PD (OR=0.03, 95%CI 0.00-0.37, *P*=0.005) were less likely to achieve nodal pCR than those who achieved CR. After completion of NAC, patients with lymph node reduction in short diameter ≥50% showed the highest possibility to achieve nodal pCR (OR=2.47, 95%CI 1.30-4.67, *P*=0.006), while patients with greater cortical thickness (OR=0.83, 95%CI 0.74-0.93, *P*=0.001) and hilum completely obliterated (OR=0.09, 95%CI 0.02-0.45, *P*=0.003) compared to hilum preservation were less likely to achieve nodal pCR.

### Nomogram development and validation

**Figure 2:**
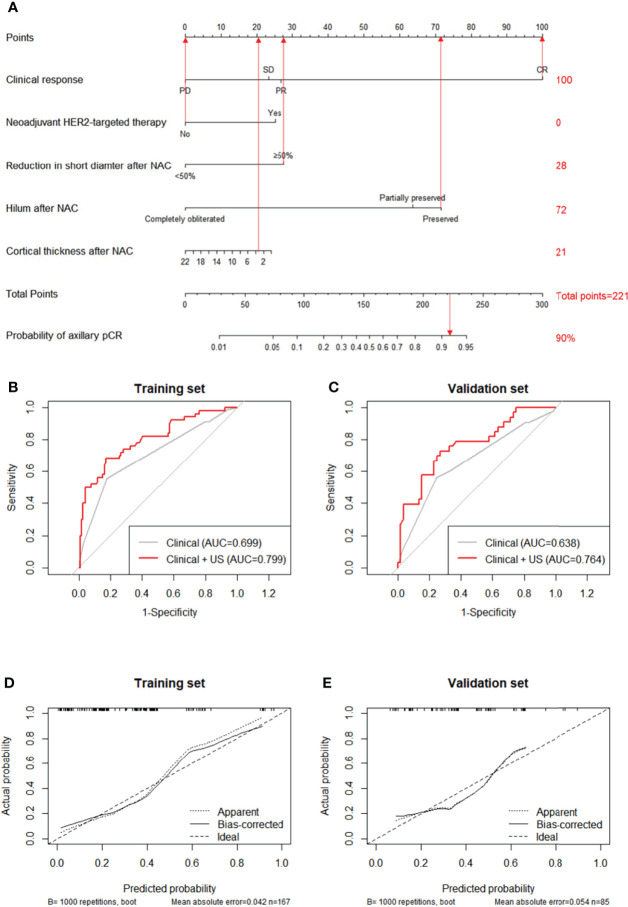
A nomogram for predicting the probability of axillary pCR **(A)**. Variables including neoadjuvant HER2-targeted therapy, clinical response, reduction in short diameter after NAC, hilum after NAC, and cortical thickness after NAC were assigned with points value. A total point added with these variables’ points indicated the probability of axillary pCR. The vertical lines between five variables and the first row can be added as a total point, the probability of axillary pCR can be finally obtained by drawing a vertical line between total points and the final row. Receiver operating characteristic curves (ROCs) of the clinical features and both clinical and US features for the prediction model in the training set **(B)**, *P*=0.001) and in the validation set **(C)**, *P*=0.027). Calibration curve of the nomogram predicting axillary pCR after neoadjuvant chemotherapy of the training set **(D)** and the validation set **(E)**.

Clinical and US variables that were statistically significant from multivariate analysis were included to construct a nomogram predicting the probability of axillary pCR (**Figure 2A**). For each patient, scores of neoadjuvant HER2-targeted therapy, clinical response, hilum status after NAC, cortical thickness after NAC, and reduction in short diameter were added up for a total pCR score from 28 to 236. The greater the pCR score, the more probable nodal pCR would be achieved.

The predictive value of the pCR score was further tested using ROC in both the training and validation set. The combined clinical and US model showed the highest AUC of 0.799 (95% CI: 0.723-0.876) in the training set, indicating the promising predictive power for nodal pCR (**Figure 2B**). Compared to the clinical-only model (AUC=0.699, 95% CI: 0.626-0.779), adding changes of US features in the model could significantly improve the diagnosis performance (0.799 *vs*. 0.699, *P*=0.001). The improvement effect of US characteristics in the combined model (AUC=0.764, 95% CI: 0.659-0.869) also confirmed in the validation set of 100 patients (0.764 *vs*. 0.638, *P*=0.027) compared to the clinical-only model (AUC=0.638, 95% CI: 0.560-0.769) as shown in **Figure 2C**. The calibration curves of the nomograms are shown in **Figure 2D** for the training set and **Figure 2E** for the validation set with 1000 steps bootstrap resampling, illustrating good consistency between the predicted result and actual probability.

## Discussion

In this study, we developed a predictive model to identify responders achieving axillary nodal pCR after NAC by combining the clinical features and changes of US features in a cohort of 296 primary node-positive breast cancer patients. Our model including neoadjuvant HER2-targeted therapy, clinical response, cortical thickness after completion of NAC, hilum status, and reduction in short diameter ≥50% after completion of NAC showed better predictive capability compared to the clinical-alone model. To our knowledge, the current study is the first to combine clinical features and changes of axillary US imaging during NAC to predict the probability of axillary nodal pCR in primary node-positive patients.

Clinical factors including neoadjuvant HER2-targeted therapy and clinical response have been commonly reported to be related to axillary nodal pCR. As expected, patients receiving neoadjuvant HER2-targeted therapy were more likely to achieve nodal pCR (28, 29). Among patients who achieved clinical CR, 60%-68% achieved nodal pCR (30, 31). Our results showed the 71% nodal pCR rate in clinical CR patients, which was consistent with the previous studies. The predicted accuracy using only clinical features of our study was in average with an AUC of 0.699, comparable with a previous study ranging from 0.649 to 0.835 (30–32).

Regarding US features, cortical thickness and complete obliteration of hilum after completion of NAC were found to be related to axillary pCR. High frequency linear array US could evaluate LNs structure such as cortex, medulla, and hilum (33). Tumor cell infiltration in LNs could cause cortical thickening, finally efface the hilum, and obscure the visualization of the hilum (16, 33). Cortical thickness can be measured as an objective and quantitative variable while status of hilum always described as a qualitative variable. Previous studies have reported that cortical thickness >3 mm after NAC is the strongest independent predictor of axillary node metastasis with an OR of 46.754 (*P*=0.000) (34). *Akissue* used cortical thickness as a continuous variable and found that longer cortical thickness was more likely to have axillary node metastasis (OR=1.84, *P*=0.005) (35). Our study corroborated these findings; longer cortical thickness was more less likely to achieve nodal pCR (OR=0.83, *P*=0.001). The absence of hilum as a later change of cortical thicken also considered to be a marker for LN metastasis. The presence of hilum was proven to be significantly associated with nodal pCR (OR=2.94, *P*=0.001) by *Huong T* (16). *Won Hwa kim* also proved that the absence of hilum was a strong predictor for lymph node metastasis (OR=14.06, *P*=0.002) (17). This result was also verified in our study; complete obliteration of hilum had the lowest OR of 0.09 (*P*=0.003) for axillary nodal pCR.

Several studies have proven that primary tumor size or tumor size change after NAC as independent characteristics associated with lymph node metastasis, indicating lymph node status as an indicator of the tumor spreading ability (12, 13, 17, 35). However, in biopsy-proven node-positive patients receiving NAC, few studies focused on the response of the lymph node itself during treatment. According to the RECIST 1.1 guideline, tumor response to treatment requires the assessment of reduction in the long diameter of the target lesions, while in the lymph node, short diameter was considered more reproducible rather than long diameter (36). Therefore, we intended to investigate the changes of lymph node US features. Our results indicated that reduction in short diameter ≥50% after NAC had an OR of 2.47 (*P*=0.006) for axillary nodal pCR.

This study aimed to evaluate the changes of axillary lymph node in order to predict axillary nodal pCR in the clinically node-positive population. US monitoring of axilla before, after two cycles, and after completion of NAC was also obtained in this current study. With the help of US techniques, nodal features can be obtained before surgery in a non-invasive, low cost, and time-saving way. Moreover, US enables us to monitor the axilla continuously at different time points of NAC, providing dynamic observation of nodal response to treatment. This concise nomogram combining the clinical and changes of US features would provide an accurate and personalized evaluation to select potential candidates who may be exempt from ALND.

There are several limitations in our study. First, this was a retrospective study which enrolled patients in a single institution. Only a limited number of patients who completed three US examinations before, during, and after NAC were included, leading to possible selection bias. Therefore, further prospective multicenter validations in larger populations are needed to verify our conclusions. Second, the largest suspicious reported node on US was chosen as the target lesion. Without special marking, the observed lymph node may not necessarily be the same, which may lead to a decrease in accuracy of our research. In our future work, we would trace the US change of lymph nodes during treatment by using potential special marking technology before NAC initiation to loce the biopsy-proven positive lymph node.

In conclusion, ultrasound feature changes during NAC could improve the accuracy of predicting node response after NAC in clinically node-positive breast cancer patients, indicating continuous US monitoring of tumor response as well as axillary lymph node feature changes would help us identify candidate patients to receive potential axilla de-escalation treatment after completion of NAC.

## Data Availability Statement

The raw data supporting the conclusions of this article will be made available by the authors, without undue reservation.

## Ethics Statement

The studies involving human participants were reviewed and approved by independent Ethical Committees of Ruijin Hospital, Shanghai Jiaotong University School of Medicine. The patients/participants provided their written informed consent to participate in this study. Written informed consent was obtained from the individual(s) for the publication of any potentially identifiable images or data included in this article.

## Author Contributions

ZL analyzed and interpreted the patient data, and was a major contributor in writing the manuscript; YT analyzed and interpreted the patient data; XC made substantial contributions to the conception of the work and substantively revised the manuscript; KS substantively revised the manuscript. All authors contributed to the article and approved the submitted version.

## Funding

The authors received financial supported from the National Natural Science Foundation of China (Grant Number: 81772797, 82072937), Shanghai Municipal Education Commission - Gaofeng Clinical Medicine Grant Support (20172007); Science and Technology Commission of Shanghai Municipality Shanghai Sailing Program (21YF1427400). All these financial sponsors had no role in the study design, information collection, data analysis or interpretation.

## Conflict of Interest

The authors declare that the research was conducted in the absence of any commercial or financial relationships that could be construed as a potential conflict of interest.

## Publisher’s Note

All claims expressed in this article are solely those of the authors and do not necessarily represent those of their affiliated organizations, or those of the publisher, the editors and the reviewers. Any product that may be evaluated in this article, or claim that may be made by its manufacturer, is not guaranteed or endorsed by the publisher.
