# Supplementary material for: Accuracy of ultrasonographic changes during neoadjuvant chemotherapy to predict axillary lymph node response in clinical node-positive breast cancer patients
Source: Front Oncol. 2022 Jul 22;12:845823. doi: 10.3389/fonc.2022.845823 (PMC9352991; doi:10.3389/fonc.2022.845823)
Supplement: Supplementary file 1 [file DataSheet_1.docx]

# Supplementary Figures

**
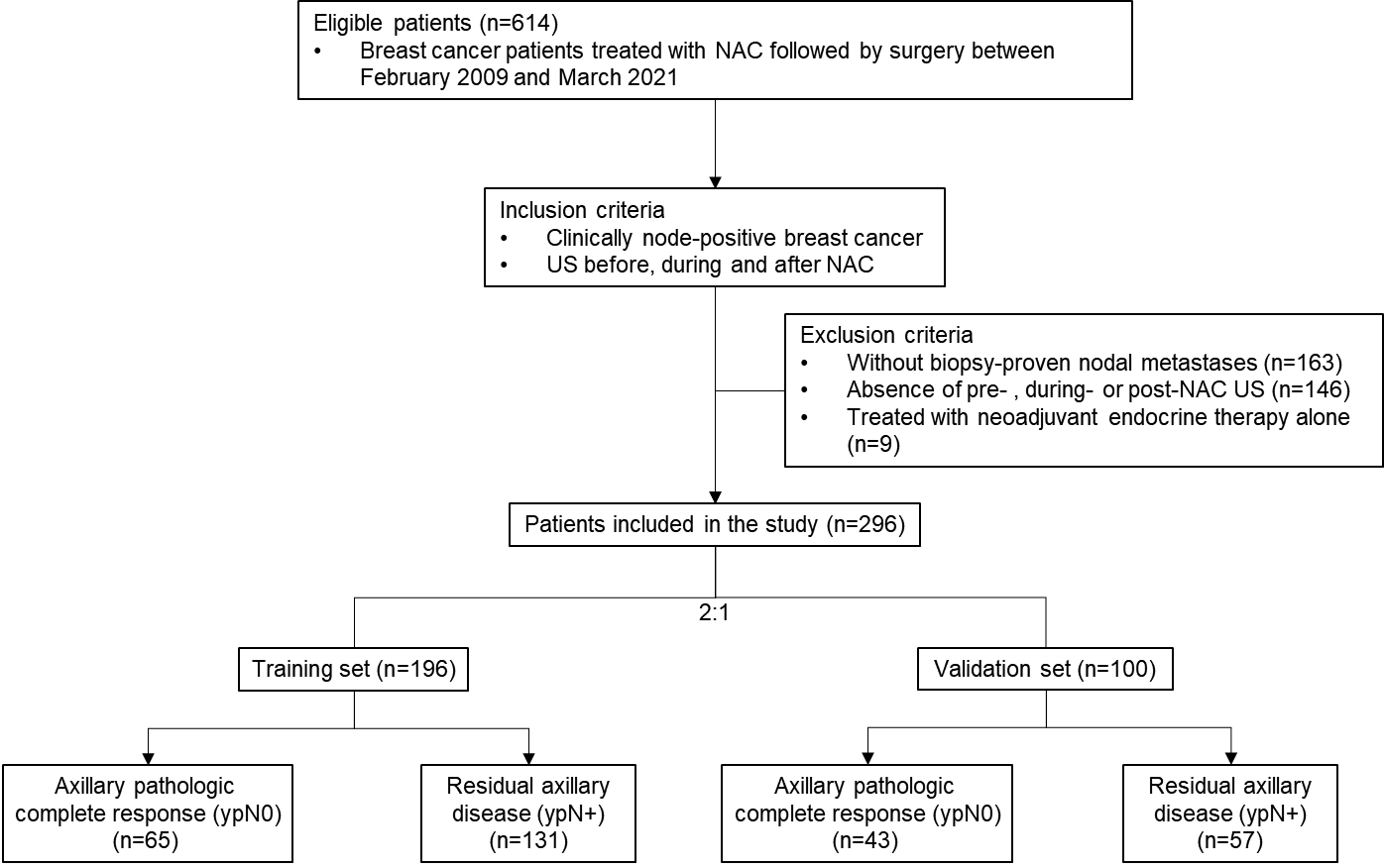
**

**Supplementary Figure 1.** Study flowchart

Abbreviations: US, ultrasound; NAC, neoadjuvant chemotherapy; ypN0, nodal pathological complete response; ypN+, residual nodal disease.

**
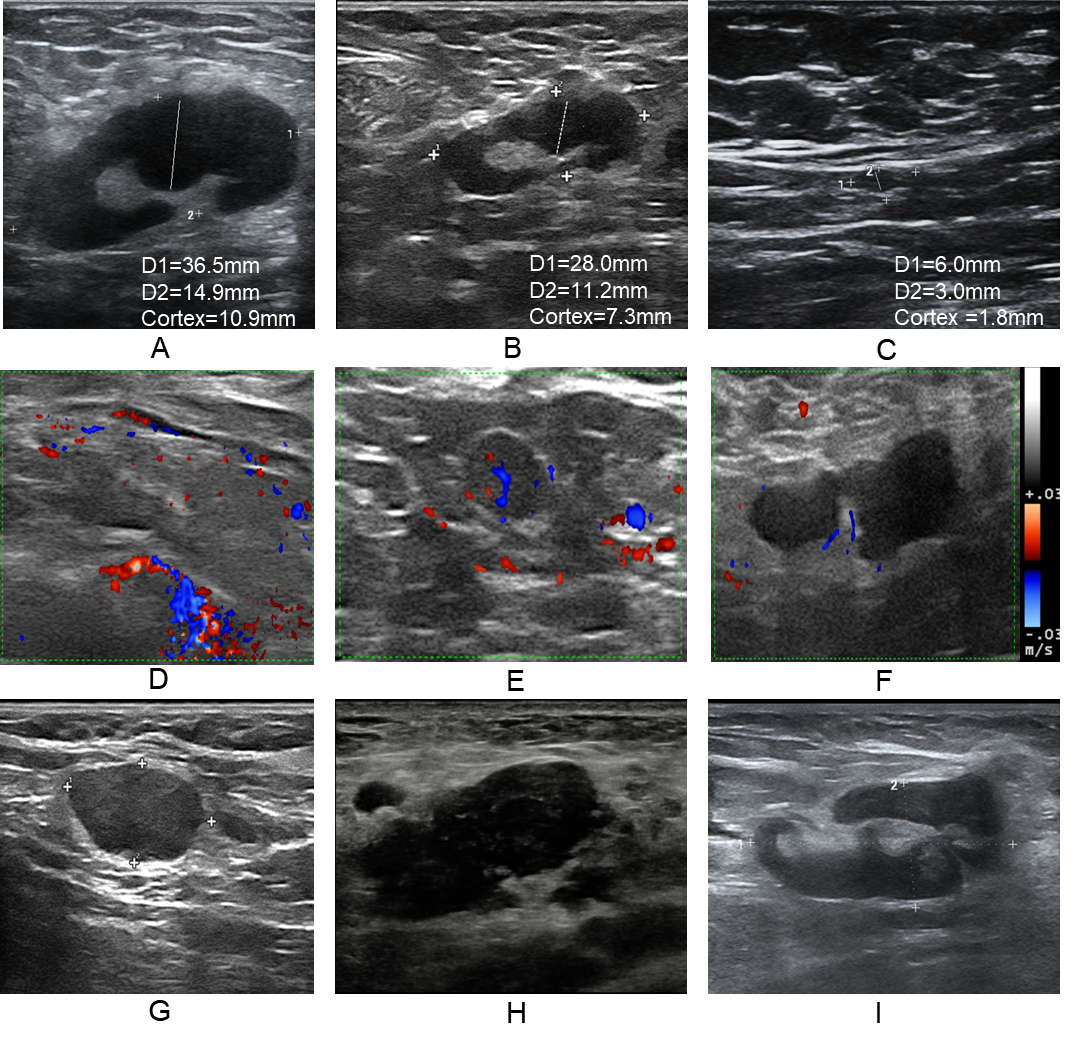
Supplementary Figure 2.** Representative ultrasound images of a biopsy-confirmed metastatic axillary lymph node at baseline (A), after two cycles of NAC (B) and after the completion of NAC (C). D1, long diameter; D2, short diameter; white line, cortical thickness. Vascularity abundant (D), minimal (E) and rare (F). Hilum completely obliterated (G), partially preserved (H) and preserved (I).

Abbreviations: NAC, neoadjuvant chemotherapy.


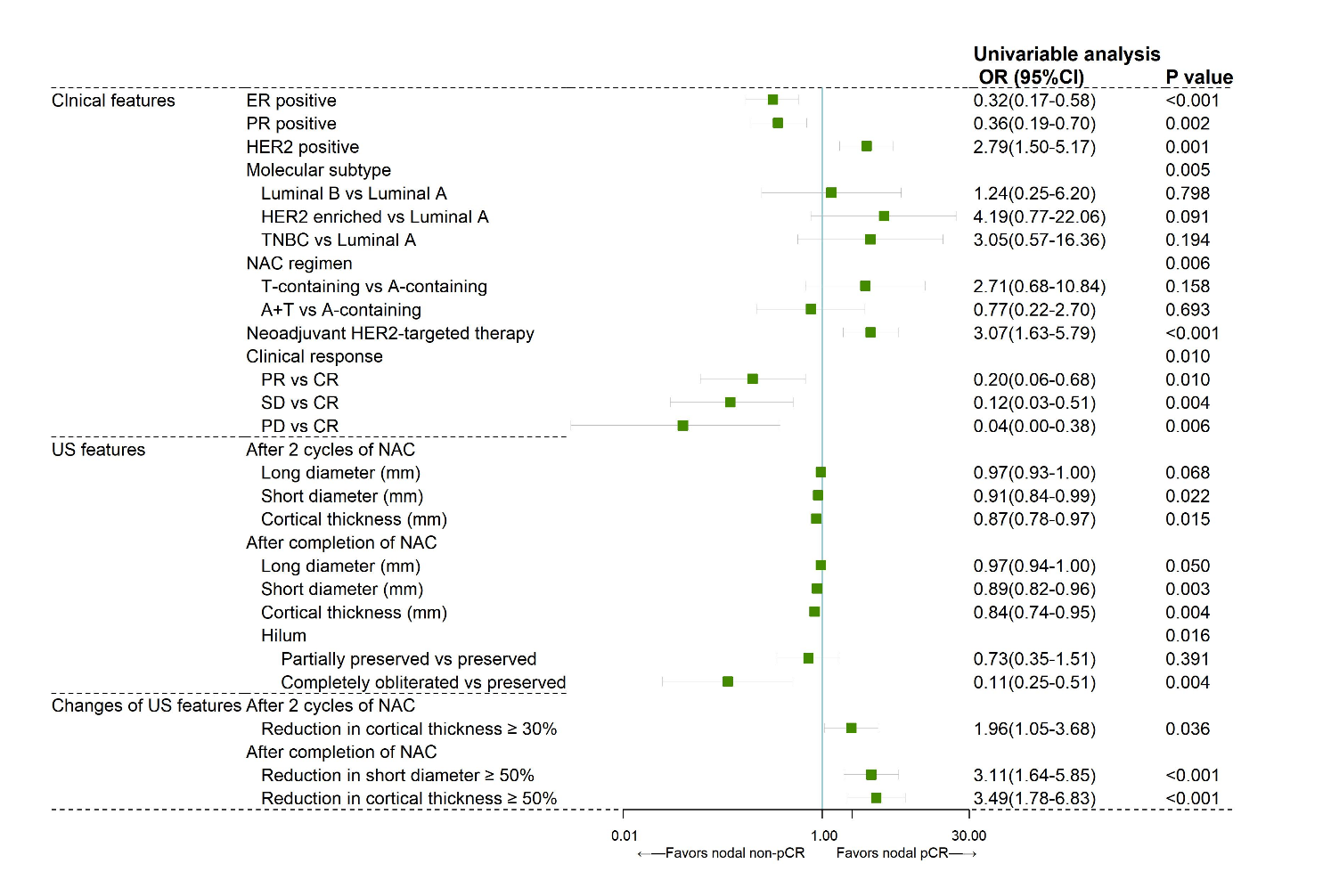
 **Supplementary Figure 3.** Results from univariable logistic regression analysis of different variables predicting axillary pCR in the training set (N=196).

Abbreviation: ER, estrogen receptor; PR, progesterone receptor; HER2, human epidermal growth factor 2; TNBC: triple negative breast cancer; A, anthracycline; T, taxanes; CR, complete response; PR, partial response; SD, stable disease; PD, progressive disease; NAC, neoadjuvant chemotherapy.
